# Reversal of end-stage heart failure in juvenile hemochromatosis with iron chelation therapy: a case report

**DOI:** 10.1186/s13256-017-1526-6

**Published:** 2018-01-26

**Authors:** Shamil D. Cooray, Neel M. Heerasing, Laura A. Selkrig, V. Nathan Subramaniam, P. Shane Hamblin, Cameron J. McDonald, Catriona A. McLean, Elissa McNamara, Angeline S. Leet, Stuart K. Roberts

**Affiliations:** 1 Department of Endocrinology & Diabetes, The Alfred Hospital, Melbourne, VIC 3004 Australia; 2 Department of Gastroenterology & Hepatology, The Alfred Hospital, Melbourne, VIC 3004 Australia; 3 Department of Advanced Heart Failure/ Transplantation, The Alfred Hospital, Melbourne, VIC 3004 Australia; 4 Membrane Transport Laboratory, QIMR Berghofer Medical Research Institute, Brisbane, QLD 4006 Australia; 5 Endocrinology & Diabetes Unit, Western Health, St Albans, VIC 3021 Australia; 6 Department of Medicine, Monash University, Melbourne, VIC Australia; 7 Department of Medicine, Melbourne Medical School – Western Precinct, The University of Melbourne, Melbourne, VIC 3021 Australia; 8 Baker Research Institute, Melbourne, VIC 3004 Australia; 9 Department of Anatomical Pathology, The Alfred Hospital, Melbourne, VIC 3004 Australia

**Keywords:** Juvenile hemochromatosis, Heart failure, Iron chelation therapy, Gene sequencing, Extracorporeal membrane oxygenation

## Abstract

**Background:**

Juvenile hemochromatosis is the most severe form of iron overloading phenotype. Although rare, it should be suspected in patients who present with hypogonadotropic hypogonadism, diabetes mellitus, or cardiomyopathy without a clear cause.

**Case presentation:**

A young Serbian male presenting with end-stage heart failure was referred for extracorporeal membrane oxygenation. An endomyocardial biopsy revealed cytoplasmic iron deposits in myocytes. His condition was stabilized with biventricular assist devices and he was listed for heart transplantation. Iron chelation therapy was commenced and resulted in rapid removal of iron burden. Serial outpatient echocardiograms demonstrated myocardial recovery such that a successful biventricular assist device explant occurred 131 days after initial implant. Targeted gene sequencing revealed a loss-of-function mutation within the *HJV* gene, which is consistent with juvenile hemochromatosis.

**Conclusions:**

This rare case of a patient with juvenile hemochromatosis associated with a *HJV* mutation provides histologic evidence documenting the reversal of associated end-stage heart failure, requiring emergent mechanical circulatory support, with iron chelation therapy.

## Background

Juvenile hemochromatosis (JH), although rare should be suspected in patients who present with hypogonadotropic hypogonadism, diabetes mellitus, or cardiomyopathy without a clear cause [1]. Targeted gene sequencing may be used to confirm this diagnosis [2]. Here we describe a remarkable case with conclusive histologic evidence documenting the reversal of the associated end-stage heart failure, requiring emergent mechanical circulatory support, previously considered to be universally fatal [1], with iron chelation therapy.

## Case presentation

A 31-year-old male was referred to a quaternary intensive care unit (ICU) for consideration for extracorporeal membrane oxygenation (ECMO) due to severe biventricular heart failure refractory to inotropic support (a cardiac index of 1.3 on noradrenaline and dobutamine infusions). He had presented with shortness of breath, extreme lethargy and abdominal pain, and was hypotensive and tachycardic.

His history was significant for a diagnosis of an iron overload state after presenting with lethargy 4 months prior. At diagnosis his serum ferritin was 2541 μg/L (reference range (RR) 30–500) with a transferrin saturation (TS) of 90% (RR 10–45). Laboratory investigations revealed normal hematologic, renal, and liver function. Hepatitis serology was negative and subsequent extensive imaging revealed no evidence of malignancy. Genetic testing revealed H63D heterozygosity for the *HFE* gene, which is inconsistent with hereditary hemochromatosis with iron overload. While awaiting venesection he developed diabetic ketoacidosis which required admission and stabilization with an insulin infusion. His glycated hemoglobin (HbA1c) was 15% and c-peptide 0.07 nmol/L (RR 0.30–2.30). He was diagnosed with diabetes mellitus secondary to iron overload with significant beta cell insufficiency and was transitioned to twice daily premixed insulin. Given the above presentation, the decision was made to proceed to liver biopsy to investigate the extent of iron overload. A liver biopsy identified extensive intrahepatocyte iron but minimal inflammatory cell infiltrate and minimal fibrosis with a hepatic iron index of 13 (RR <2). His liver function tests were normal.

Our patient, of Serbian origin, reported no family history of iron overload syndrome, endocrinopathy or cardiomyopathy. There was no history of excess alcohol intake, recreational drug use, or toxic environmental exposures.

A physical examination on admission to the ICU revealed a patent airway. His respiratory rate was 15 breaths/minute, oxygen saturation 98% on 4 liters of oxygen via nasal prongs, and good air entry to bilateral lung fields but slightly reduced at the right base without crackles or wheeze. On a noradrenaline, dobutamine, and amiodarone infusion his blood pressure was 90/64 mmHg with a pulse rate of 105 beats per minute with cardiac monitoring revealing a sinus tachycardia. A cardiovascular examination was significant for a hyperdynamic apex beat with heave, a jugular venous pulse at 2–3 cm and dual heart sounds with nil murmurs. A neurological examination was significant for a Glasgow coma score of 14 (eyes 3 motor 5 verbal 6). His abdomen was soft on palpation with tenderness to the right upper and lower quadrant and no guarding. A peripheral examination was significant for a tanned complexion and the absence of peripheral edema.

Laboratory investigations on admission did not account for the patient’s critical clinical state revealing normal hemoglobin concentration, platelet count, renal function and serum electrolytes. His liver function was mildly deranged with an elevated alanine transaminase (88 units/ L, RR 12–15) and bilirubin (62 umol/L RR, ≤ 23) and decreased albumin (30 g/L, RR 33–46). His C-reactive protein (CRP) and neutrophil count were mildly elevated, 9 mg/L (RR ≤ 5) and 10.27 10^9^/L (RR 1.90–8.00) respectively, with a normal white blood cell count (12.03 10^9^/L, RR 3.90–12.70). Antinuclear antibody and rheumatoid factor test results were negative. A high-sensitivity Troponin I test result was mildly elevated (64 ng/L, RR ≤ 26). Midstream urine and blood culture were negative.

A computed tomography scan demonstrated a dilated heart with associated pleural effusions, ascites, and liver congestion. A transthoracic echocardiogram revealed normal left ventricular (LV) size with severe global systolic dysfunction with an ejection fraction of 5–10%, spontaneous echo contrast, and normal wall thickness. The right ventricular (RV) size was normal with severely reduced function. Mild to moderate mitral and pulmonary regurgitation were noted along with a trivial pericardial effusion.

**Fig. 1:**
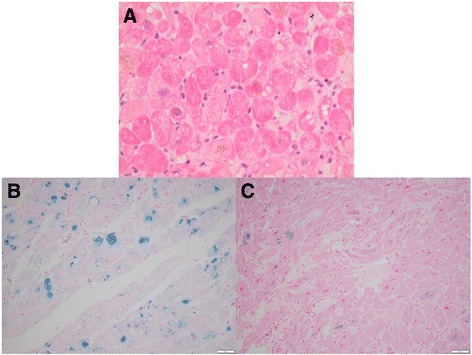
Endomyocardial biopsy tissue showing features of hemochromatosis. **a** Initial biopsy showing hypertrophic muscle fibers and abundant intracytoplasmic pigment with variable vacuolization; hematoxylin and eosin stained ×400 actual magnification. **b** Intracytoplasmic ferric iron confirmed with Perls’ Prussian blue stain; ×200 actual magnification. **c** Repeat biopsy 141 days later demonstrates marked reduction in ferric iron content following iron chelation therapy; Perls’ Prussian blue stain ×200 actual magnification

He had a prolonged ICU stay due to progressive multi-organ dysfunction. He was intubated and ECMO support was initiated on day 2 and biventricular assist devices (BiVAD) were inserted on day 8. An endomyocardial biopsy revealed myocyte intracytoplasmic iron deposition (Fig. 1a and b). This result together with the global nature of the systolic dysfunction was consistent with iron overload as a cause for the cardiomyopathy. There was no echocardiographic or histopathologic evidence to suggest myocarditis and hemodynamic instability precluded coronary angiography.

Serum ferritin (SF) level was 12,361 μg/L and TS was 99% on day 2. Iron chelation therapy was commenced on day 6 with desferrioxamine, initially subcutaneously. Shortly after its commencement, increasing inotropic requirements and bilateral lung infiltrates felt to represent pulmonary edema were noted and desferrioxamine was ceased as a precaution. It was restarted on day 21 once his clinical condition had stabilized and up-titrated to a maximum dose of 4200 mg (60 mg/kg) administered via a 24-hour intravenous infusion and was subsequently well tolerated with rapid removal of iron burden. SF levels peaked on day 13 at 18,676 μg/L and TS was > 99% before decreasing to 1055 μg/L by day 59 with TS also decreasing to 65%.

By the fifth week after presentation, it was considered that he may have developed diabetes insipidus on the basis of a marked unexpected increase in urine output, but because of his parlous clinical state on BiVAD it was not possible to confirm with a water deprivation test at that time. A trial of desmopressin did not result in convincing improvement.

Investigation of his anterior pituitary function revealed isolated hypogonadotropic hypogonadism. Testosterone replacement was commenced with testosterone 1% gel 2.5 g topical daily with subjective improvement in energy levels and sense of wellbeing.

Our patient was listed for heart transplantation and discharged home on day 69 with BiVAD in situ after progressive improvement in his physical capacity with intensive inpatient rehabilitation. Desferrioxamine was continued at home via nightly 10-hour continuous subcutaneous infusions. Our patient was readmitted on day 89 with urosepsis, which was successfully treated with antibiotic therapy. At this time, he was noted to have improved left ventricle function on an echocardiogram, estimated to be only mildly reduced on a technically difficult study. Serial outpatient echocardiograms, both resting and stress, demonstrated myocardial recovery with normal LV size and resting systolic function (visual LV ejection fraction of 55–60%) and normal RV size and low-normal systolic function. With exercise, there was good augmentation of both LV and RV function and a normal hemodynamic response. Successful BiVAD explant occurred, 141 days after initial implant with SF 163 μg/L and TS <1% 12 days prior. Left ventricular biopsy at this time demonstrated less intracytoplasmic iron deposition compared with the initial biopsy (Fig. 1c). Post BiVAD explant, desferrioxamine was ceased as a potential cause of worsening renal tubular acidosis. Cardiac magnetic resonance imaging (cMRI) completed 2 weeks post BiVAD explant demonstrated normal biventricular systolic function but a myocardial T2* time of 12.3 milliseconds (normal > 20 milliseconds) consistent with residual myocardial iron loading. A formal water deprivation test was performed and was normal, therefore desmopressin was ceased.

As the patient’s history was significant for a diagnosis of hemochromatosis, JH was suspected given his age and absence of family history. Targeted gene sequencing was then performed across 39 genes and 11 promoter regions using a custom “AmpliSeq” panel (Life Technologies, Mulgrave, Victoria, Australia) and an Ion Torrent™ Personal Genome Machine™ (Life Technologies) as previously described [2]. Following alignment of the sequencing with the Human Genome version 19 (HG19), our patient’s heterozygosity for the *HFE* H63D was confirmed, and no other mutation were present in the *HFE* gene. Variant analysis identified homozygosity for a variant c.G959T (NM_213653) in exon four of the *HFE2* gene (aka *HJV*) resulting in a p.G320V mutation (rs74315323). This DNA change was confirmed by Sanger sequencing. The G320V mutation has previously been reported in multiple cases of juvenile onset hereditary hemochromatosis [3]. No other mutations known to be associated with iron overload were identified in our patient, nor were there any other novel or rare single nucleotide polymorphisms in the coding regions of sequenced genes.

At follow-up 12 months after his presentation with severe biventricular heart failure the patient has made a near-complete recovery and had returned to work. His SF level was 29 μg/L. CMRI, previously contraindicated due to the implanted ventricular assist devices, 20 months after he presented with severe biventricular heart failure, demonstrated T2* time had returned to normal in the myocardium (41 milliseconds) and liver (19 milliseconds). The study also demonstrated normal biventricular function with mild biventricular dilatation and the absence of myocardial fibrosis. He remains on insulin and testosterone replacement and 3-monthly venesections were commenced.

## Discussion

This case demonstrates that although rare, juvenile hemochromatosis should be suspected in patients who present with hypogonadotropic hypogonadism, diabetes mellitus, or cardiomyopathy without a clear cause. It also illustrates how targeted gene sequencing may be used to confirm the diagnosis. Finally, to the best of our knowledge, this case report is the first to document conclusive histologic evidence of the reversal of the associated end-stage heart failure, requiring emergent mechanical circulatory support, with iron chelation therapy.

While the H63D variant in *HFE* has a high global prevalence with a 15% allele frequency in Europeans and South Americans, it is not usually clinically associated with iron overload. It may, however, affect iron loading when in a compound heterozygous state with the C282Y mutation [4, 5].

Juvenile hemochromatosis (JH) or type 2 hereditary hemochromatosis, is a rare condition affecting about 1 in 4.8 million people [6] and results from autosomal recessive loss-of-function mutations in either the *HJV* (type 2a) or *HAMP* (type 2b) genes. Mutations within *HJV* account for almost all JH, with only a handful of *HAMP* variant-related cases ever reported.

HJV is a key receptor in the iron regulatory pathway, transducing iron sensing signals to the iron-regulatory peptide hepcidin, which then leads to the restriction of iron absorption and recycling, preventing iron overload. To date, more than 50 loss-of-function mutations have been reported within the *HJV* gene associated with JH [7]. Of these, the *G320V* variant is the most common and has been reported across a diverse range of regions with strong European ancestry, including Australia [7].

As an acute phase reactant, ferritin levels are increased by inflammatory cytokines [8] and thus the positive predictive value of an elevated isolated SF for hemochromatosis may be as low as 18% [9]. TS is a useful adjunct as an elevated value suggests iron overload, as in this case. Conversely, hyperferritinemia with a normal TS usually suggests a reactive cause [10]. In this case the marked increase in ferritin to extremely high levels (>3000 μg/L) at the time of presentation likely reflects widespread cellular injury as a result of the patient’s critical clinical condition over and above that reflective of increased ferritin synthesis due to underlying iron overload [11, 12]. Iron studies may also suggest the degree of iron overload and the likelihood of organ damage. SF levels greater than 1000 μg/L predicts liver damage in patients with hemochromatosis [13]. Similarly, TS exceeding 85% are thought to correspond with an increased risk of cardiac and endocrine iron accumulation due to the circulation of non-transferrin-bound iron (NTBI) [14]. NTBI is toxic and is readily taken up by hepatocytes and cardiomyocytes and thus may be a direct marker of iron toxicity but this was not able to be assessed in this case [15].

Clinically, JH results in the most severe form of iron loading phenotype, with SF typically significantly greater than 1000 μg/L and TS of 80–100% before the age of 30 years, as in this case [1]. Furthermore, multi-organ iron loading is common, and as a result of this, other presenting symptoms may include hypogonadotropic hypogonadism or associated reproductive problems, arthropathy, diabetes mellitus, and cardiomyopathy [1, 7]. Together, these symptoms can lead to significant morbidity, with heart failure being the leading cause of death amongst JH cases [1]. Reversal of heart failure is rare, reported in a case of presumed juvenile hemochromatosis but without confirmatory genetic analysis [16], associated with a mutation in the *HAMP* gene [17] and most recently in association with a *HJV* mutation [18] like in our case but in all with less advanced heart failure with LV ejection fraction > 20% as compared to 5–10% requiring emergent mechanical circulatory support as in this case.

Iron overload due either to excess gastrointestinal iron absorption or secondary to transfusion load is a well-recognized cause of reversible cardiomyopathy, and is a leading cause of mortality in the thalassemia patient group, and a rare indication for heart transplantation [19–21]. A long asymptomatic phase, during which time there is cardiac iron deposition without cardiac dysfunction, is followed by acute clinical and imaging deterioration relating to the iron storage capacity being exhausted and the formation of NTBI, which is toxic to the myocytes [19, 21]. SF and liver iron stores do not correlate with cardiac iron accumulation. cMRI with T2* imaging has provided a noninvasive quantitative assessment of cardiac iron stores [19, 21]. The long asymptomatic phase and poor correlation with other clinical markers in conjunction with the reversible nature of the condition highlight the importance of specific assessment for cardiac iron overload in patients with increased burden of iron [19].

Treatment with chelation agents such as desferrioxamine have demonstrated reduced iron load in the heart, as assessed by T2* imaging, and improved systolic function [22] as demonstrated in this case. Furthermore, to the best of our knowledge, this is the first case to provide conclusive histologic evidence that desferrioxamine reduces iron load in the heart, reversing severe cardiac hemochromatosis with resultant normalization of cardiac function. Desferrioxamine therapy has been extensively studied in transfusion-dependent patients with thalassemia and has been shown to decrease myocardial iron content by about 24% [23], reverse early cardiac hemochromatosis [22], improve LV function [24] and prolong survival [25]. This case demonstrates that monotherapy with this agent is efficacious and avoids the serious risks of neutropenia associated with oral iron chelator, deferiprone [26], which has been suggested could be used in combination with desferrioxamine for more intensive chelation [27]. Where mechanical support is available as in this case, monotherapy may be a more appropriate option to reverse end-stage heart failure.

This case reaffirms the premise that myocardial iron loading is a reversible cause of heart failure even when at its most severe and, where available, support with extracorporeal or assisted circulation can provide the time required for chelation to occur. To this date such a significant clinical, echocardiographic and histologic improvement from single-agent chelation therapy, as achieved in this case, is yet to be documented.

## Conclusions

Juvenile hemochromatosis is the most severe form of iron overloading phenotype. Although rare, it should be suspected in patients who present with hypogonadotropic hypogonadism, diabetes mellitus, or cardiomyopathy without a clear cause. The end-stage heart failure associated with juvenile hemochromatosis, previously considered to be universally fatal, might be reversible with chelation therapy.

## Abbreviations

BiVAD: Bi-ventricular assist devices
cMRI: Cardiac magnetic resonance imaging
CRP: C-reactive protein
ECMO: Extra-corporeal membrane oxygenation
HbA1c: Glycated hemoglobin
HG19: Human Genome version 19
ICU: Intensive care unit
JH: Juvenile hemochromatosis
LV: Left ventricular
NTBI: Non-transferrin-bound iron
RR: Reference range
RV: Right ventricular
SF: Serum ferritin
TS: Transferrin saturation
